# Low and then high frequency oscillations of distinct right cortical networks are progressively enhanced by medium and long term Satyananda Yoga meditation practice

**DOI:** 10.3389/fnhum.2014.00197

**Published:** 2014-06-10

**Authors:** John Thomas, Graham Jamieson, Marc Cohen

**Affiliations:** ^1^School of Health Sciences, RMIT UniversityBundoora, VIC, Australia; ^2^School of Behavioural, Cognitive and Social Sciences, University of New EnglandArmidale, NSW, Australia

**Keywords:** meditation, Yoga, EEG, eLORETA, neural networks, default mode network

## Abstract

Meditation proficiency is related to trait-like (learned) effects on brain function, developed over time. Previous studies show increases in EEG power in lower frequency bands (theta, alpha) in experienced meditators in both meditation states and baseline conditions. Higher gamma band power has been found in advanced Buddhist meditators, yet it is not known if this occurs in Yoga meditation practices. This study used eLORETA to compare differences in cortical source activity underlying scalp EEG from intermediate (mean experience 4 years) and advanced (mean experience 30 years) Australian meditators from the Satyananda Yoga tradition during a body-steadiness meditation, mantra meditation, and non-meditation mental calculation condition. Intermediate Yoga meditators showed greater source activity in low frequencies (particularly theta and alpha1) during mental calculation, body-steadiness and mantra meditation. A similar spatial pattern of significant differences was found in all conditions but the number of significant voxels was double during body-steadiness and mantra meditation than in the non-meditation (calculation) condition. These differences were greatest in right (R) superior frontal and R precentral gyri and extended back to include the R parietal and occipital lobes. Advanced Yoga meditators showed greater activity in high frequencies (beta and especially gamma) in all conditions but greatly expanded during meditation practice. Across all conditions (meditation and non-meditation) differences were greatest in the same regions: R insula, R inferior frontal gyrus and R anterior temporal lobe. Distinct R core networks were identified in alpha1 (8–10 Hz) and gamma (25–42 Hz) bands, respectively. The voxels recruited to these networks greatly expanded during meditation practice to include homologous regions of the left hemisphere. Functional interpretation parallels traditionally described stages of development in Yoga proficiency.

## Introduction

The introduction of Yoga and Buddhist meditation practices into Western countries has extended their application beyond their original spiritual goals (Shapiro, 2003) to include a range of mind-body interventions for health-related problems (Ospina et al., 2007). Research has explored the neurophysiology of meditation during the actual practice (as examples of specific altered states of consciousness) and changes persisting into non-meditation conditions (as examples of neuroplasticity). A central question in this investigation is how the level of meditator proficiency contributes to the development of these effects.

As yet there is no accepted objective measure of meditator proficiency. Most studies report proficiency in years, although a more recent trend uses the more accurately calculated “hours of practice,” “based on daily practice and time spent in meditative retreats” (Brefczynski-Lewis et al., 2007). The term “advanced” has usually been reserved for meditators with more than 20 years' experience (Arambula et al., 2001). Numerous studies conducted over the last 40 years with Western meditators, usually with less than 10 years' experience, have reported increased power and coherence in the alpha and theta frequency bands during meditation practice (Cahn and Polich, 2006). Striking increases in gamma band power have also been reported in studies with “advanced” Buddhist monks as meditators (Lehmann et al., 2001; Lutz et al., 2004). A recent study also found significantly increased gamma power at parieto-occipital electrodes in a group of “advanced” Western Vipassana meditators (with mean 20 years' meditation experience—although the nature of that experience is undefined) engaged in a “mindfulness” body-scan meditation when compared to a deliberate mind-wandering instruction condition (Cahn et al., 2010).

Fell and colleagues proposed that meditators from different traditions progress through similar developmental stages, which are marked by changing EEG frequency patterns (Fell et al., 2010). Initial expertise is reflected in changes in slower (specifically theta and alpha) frequency bands, but these effects are not considered to be meditation-specific. The authors propose that an “advanced” stage, only reached by experts, is marked by increased synchronized (fast) gamma band activity, related to “processes of cortical restructuring and learning” which facilitate “specific meditation-related states of consciousness,” with unique electrophysiological signatures.

A recent focus in EEG and brain imaging meditation studies has been on the pivotal role of the modulation of the “default mode network” (DMN) in the understanding of meditation training effects (Brewer et al., 2011; Berkovich-Ohana et al., 2012; Malinowski, 2013). This neural network is considered to be active when effortful attention is not required to direct responses to the external environment. Anatomically the DMN consists of two major interacting subsystems defined by the cluster of activations and functional connections around medial prefrontal and medial parietal hubs, respectively (Buckner et al., 2008). A series of both EEG and fMRI studies have converged on the finding of reduced activity in these major hubs of the DMN during Buddhist and/or mindfulness meditation practice (Farb et al., 2007; Hölzel et al., 2007; Berkovich-Ohana et al., 2012). The DMN is known to be activated by spontaneous self-related mentation, directed to either the future or the past (Schooler et al., 2011), characteristic of distraction or mind wandering. Both Yoga and Buddhist meditation traditions^1^ note the emergence and progressive resolution of distraction and mind wandering during the early phases of mediation training irrespective of content. Rather than belonging to the essence of states cultivated by respective meditation practices, current DMN findings may only reveal the expectable psychological effects of early steps in the process of learning to meditate.

Recent brain imaging techniques have begun to reveal training effects of a variety of meditations, across a range of practice periods, on the neuroplastic response of the brain in both gray matter and white matter structures (Lazar et al., 2005; Pagnoni and Cekic, 2007; Holzel et al., 2008, 2011; Luders et al., 2009, 2011, 2012a,b; Vestergaard-Poulsen et al., 2009; Grant et al., 2010; Tang et al., 2010; Murakami et al., 2012; Kang et al., 2013; Leung et al., 2013). In EEG studies Berkovich-Ohana et al. (2012) found significantly greater gamma-band activity at rest in mindfulness meditators than controls in a cluster of electrodes in the right parieto-occipital region and the reverse in a cluster of electrodes in the right frontal region, however no significant relationship was reported for these measures with meditation experience within the mindfulness meditation group. Cahn et al. (2010) found gamma power at occipital electrodes increased significantly from rest to “vipassana meditation” in those with greater than 10 years of daily practice but only marginally in those with less than 10 years of daily practice. Although their findings vary widely (as might be expected from their methodological differences) these studies point to the importance of evaluating both the long-term impact of specific meditation practices and the way in which that impact unfolds over time. Understanding these medium and longer term changes may require a focus beyond the role of the DMN.

Within the Yoga tradition sequential stages in the development of meditation proficiency are delineated in the “eight-fold” path of Patanjali's Yoga Sutras (Radhakrishnan, 1999). Following four “external” stages, the practitioner progresses through the “internal” stages of “*pratyahara*” (sense withdrawal), “*dharana*” (concentration), “*dhyana*” (absorption), and finally “*samadhi*” (self-realization). Repeated experience of these states during meditation practice leads to a long-term progressive refinement in the “sense of self” (*asmita*). One's self-identity becomes progressively detached from identification with externally-oriented perceptions, then from identification with the body, then from identification with thoughts, to absorption in the object of meditation in *samadhi*. The process has been likened to peeling the skins from an onion (Maheshwarananda, 2005). However, the electrophysiological changes corresponding to such a developmental sequence will not be evident within current cross-sectional designs comparing only short-term and non-meditators or short-term and long-term meditators. While longitudinal studies are ideal, as a first step it is necessary to track the transition between medium term and long term effects of Yoga meditation training.

The present study made use of the long period of establishment of Satyananda Yoga in Australia for the availability of “advanced” Western practitioners with over 30 years' experience. The study compared “advanced” Australian Satyananda Yoga teachers (SYT) with students studying to become SYT (having an intermediate level of experience). Two meditation practices were used for the study. The first “*kaya sthairyam*” is a preparatory body-steadiness practice designed to take the practitioner into the “*pratyahara*” stage (withdrawal of the mind from the external world). The second practice “*japa*” uses mental repetition of a personal mantra to move from “*pratyahara*” to the “*dharana*” stage (a focussed internal awareness of the mantra). Advanced practitioners may progress further to “*dhyana*” (absorption in the mantra) or even “*samadhi*” in this practice (Saraswati, 1983). Based on this tradition, we hypothesized that student meditators are more likely to experience sense withdrawal; the advanced practitioners are more likely to progress to the later stages when engaged in these practices.

In keeping with results from moderately experienced Western meditators and the demands of sensory inhibition, we hypothesized firstly that the Satyananda students (with an intermediate level of meditation practice) would show more EEG activity in the lower frequency bands (theta, alpha1, and alpha2). It was expected that this activity would be higher in the students than in the SYT group as a consequence of the teachers' development beyond this stage of practice. Secondly, it was expected that the SYT would show greater activity in the higher frequency bands (beta and gamma) compared to the Satyananda Yoga student group in both meditation conditions. Thirdly, extended practice (spanning years) in both student and teacher groups was expected to result in enhanced neural connectivity and thus trait activation in networks habitually activated in each group during meditation outside the context of meditation itself. Fourth and finally, to the extent that Yoga meditation practices engender similar DMN changes to the Buddhist-mindfulness practices recently investigated by meditation researchers, it was expected that the cortical foci of these length of practice differences would lie in the anterior and posterior midline hubs of the DMN at least during active meditation practice^2^.

## Materials and methods

### Participants

Twelve Satyananda Yoga practitioners (teachers and students) were recruited for the study from the Satyananda Yoga Academy in Mangrove Creek, NSW, Australia by the first author. At the time of the study, he was a resident lecturer at the Academy. In his sixties, he is a *karma sannyasin* disciple of Swam Satyananda of Munger, India. Participants were recruited initially by direct contact for a qualitative study of meditation. Following that study, participants for the EEG study were selected from suitable volunteers. All the teachers had received initiation as *sannyasin* disciples of Swami Satyananda and had been regular meditators for over 20 years. The student group was studying an accredited course to qualify as SYT. All participants had received a personal mantra from their guru (Swami Satyananda or his successor, Swami Niranjanananda), which they used in this study. In pre-study interviews, all reported they were free from medical, psychiatric, or drug usage issues that might alter their brain functioning.

The participants were divided into two groups—SYT, three male and three female, age range from 44 to 63 years (mean = 54 years, *SD* = 6.5 years) and Satyananda Yoga students (SYS), three male and three female trainee Yoga teachers, age range 30–51 years (mean = 42 years, *SD* = 8.0 years). The teacher group was significantly older [*t*_(11)_ = 2.90, one-tailed, *p* < 0.05]. The SYT group had a mean of 30 years regular practice (range: 24–37 years) and the SYS group a mean of 4 years (range: 3–5 years). Based on an average of 1 h regular practice a day, this would equate to a mean of 11,000 h for the SYT group and 1500 h for the SYS group.

### Setting

The study was conducted in a small meditation room at the Satyananda Yoga Academy to provide an “ecologically valid” situation conducive to the attainment of deep meditation states. After being fitted with the Compumedics 32 channel EEG “Quik Cap,” participants sat in their usual cross-legged meditation position on the floor, supported by cushions, with the room dimly lit by a candle on a meditation table. The small battery-operated Compumedics “Siesta 802” recording unit attached to the “Quik Cap” was the only electronic device in the meditation room. This unit transmitted signals by radio to a laptop computer in an adjacent room.

### Procedure

To obtain the most authentic meditation experience possible, the sequence of conditions was selected to resemble the practitioners' usual meditation practices. We considered that a design incorporating a counterbalanced order of conditions would introduce a conflict with traditional practice, as *kaya stairyam* would usually precede but not follow *japa* (Saraswati, 1981). Following the mental calculation condition, the participants performed four meditation practices in the same order (for brevity only the first two of these are reported here).

The experimental conditions were:

- Non-meditation condition—“Calculation”—mentally counting backwards from 200 by 4 s—(5 min).
- Meditation 1—“Body-steadiness”—Satyananda Yoga practice “*kaya stairyam*”*—*awareness focused on body-steadiness and awareness of flow of the natural breath—(5 min).
- Meditation 2—“Mantra”—Satyananda Yoga practice “*japa*”—mental repetition of personal mantra, using mala (beads). The mantra consisted of a short Sanskrit phrase—(10 min) (Saraswati, 1981).

All conditions were performed, sitting in cross-legged meditation posture, with eyes closed. Self-report ratings of “meditation depth” for each condition were obtained immediately following the EEG recording. These ratings were made on a ten-point visual analog scale, based on the “Meditation Depth Questionnaire” of Ulrich Ott (2001). The zero point was “no meditation state” and ten was “deepest meditation I have experienced.”

The study was approved by the RMIT University Human Research Ethics Committee.

### EEG data collection

EEG signals were obtained using a Compumedics “Quik Cap” from 25 scalp electrodes, based on the International 10/20 system, referenced to left mastoid. Electrodes were placed at FP1, FP2, F3, F4, C3, C4, P3, P4, O1, O2, F7, F8, T3, T4, T5, T6, Fz, Cz, Pz, Oz, TP7, TP8, CP3, CP4, FC3. Four additional channels were allocated to eye movement detection, with electrodes positioned on the outer canthi of each eye and above and below the left eye. The sampling rate was 256 Hz. Data was acquired via radio signal to a laptop computer running Compumedics Profusion EEG software.

### EEG data pre-processing

Following the recording session, the data was exported from the Profusion EEG in EDF format for input into the EEGlab (Delorme and Makeig, 2004) program running in Matlab. Data was preprocessed through the FASTER algorithm (Nolan et al., 2010) which employs independent components analysis (ICA) to identify and remove both physiological (eye movements/blinks, muscle movement, skin potentials) and non-physiological (electromagnetic interference, electrode pop offs and drift, shifting electrodes and residual white noise) sources of artifact from the recorded EEG. FASTER interpolated missing or bad channels, re-referenced to the common average and applied a bandpass filter of 1–45 Hz to remove drift and further high frequency artifacts. FASTER detects and removes ICA components with properties uncharacteristic of cortical signals or conversely with properties characteristic of specific artifact sources. Z-score thresholds for rejecting artifactual components were set at 3.0 (except for eye movement, which was set at a threshold of 1.8). EEGLAB version 9 (Delorme and Makeig, 2004) was employed for criterion-based artifact rejection of epochs with values greater than ±75 mV. The recorded EEG was then subject to visual inspection as a final check of artifact removal.

Ten sample epochs, each of 5 s duration, were extracted for the first 5 min of each condition for each participant from the cleaned datasets, commencing 100 s from the start of the practice and then at 20 s intervals. If a selected epoch showed residual artifacts on visual inspection, the subsequent 5 s epoch was selected. The epochs were analyzed using a user defined frequency allocation into bands of: delta (1–4 Hz), theta (4–8 Hz), alpha1 (8–10 Hz), alpha2 (10–12 Hz), beta (12–25 Hz), and gamma (25–42 Hz).

Precautions were taken to ensure the gamma band analysis was not confounded by electromyographic (EMG) muscle activity or eye-saccades artifacts. All conditions were conducted with eyes-closed. Each epoch was visually inspected for artifacts and the cut-off frequency for gamma was set well below the EMG frequency range, which peaks at 70–80 Hz (Lutz et al., 2004).

### EEG source analysis

Based on the scalp-recorded electric potential distribution, the exact low resolution brain electromagnetic tomography (eLORETA) software (publicly available free academic software at http://www.uzh.ch/keyinst/loreta.htm) was used to compute the cortical three-dimensional distribution of current source density (CSD). The eLORETA method is a discrete, three-dimensional (3D) distributed, linear, weighted minimum norm inverse solution. The particular weights used in eLORETA endow the tomography with the property of exact localization to test point sources, yielding images of current density with exact localization but low spatial resolution (neighboring neuronal sources will be highly correlated). The description of the method together with the proof of its exact zero-error localization property, are described in two papers by Pascual-Marqui (2007, 2009). It is important to note that eLORETA has no localization bias even in the presence of structured noise which constitutes an improvement over the previous tomographies of LORETA (Pascual-Marqui et al., 1994) and the standardized version sLORETA (Pascual-Marqui, 2002). It is important in the context of assessing length of practice related differences in anterior and posterior hubs of the DMN that activity in these deep structures can be correctly localized with these methods (Pizzagalli et al., 2001; Zumsteg et al., 2006).

Current eLORETA computations were made using a realistic head model (Fuchs et al., 2002), using the MNI152 template (Mazziotta et al., 2001), with the three-dimensional solution space restricted to cortical gray matter, as determined by the probabilistic Talairach atlas (Lancaster et al., 2000). Standard electrode positions on the MNI152 scalp were taken from Jurcak et al. (2007) and from Oostenveld and Praamstra (2001). The intracerebral volume is partitioned in 6239 voxels of 5 × 5 × 5mm^3^ spatial resolution. These eLORETA images represent the electric activity at each voxel in Montreal Neurological Institute (MNI) space as the exact magnitude of the estimated current density. Anatomical labels and/or Brodmann areas are reported using MNI space, with correction to Talairach space (Brett et al., 2002).

The KEY Institute eLORETA software package was used to perform these statistical analyses. The methodology used is non-parametric. It is based on estimating, via randomized permutation testing, the empirical probability distribution for the value of the maximum statistic across all voxels under the null hypothesis. This methodology corrects for multiple testing (i.e. for the collection of tests performed for all voxels, and for all discrete frequencies). Due to the non-parametric nature of the method, its validity need not rely on any assumption of Gaussianity. The reader is referred to Nichols and Holmes (2002) for a detailed overview of this methodology.

## Results

### Subjective ratings

**Figure 1:**
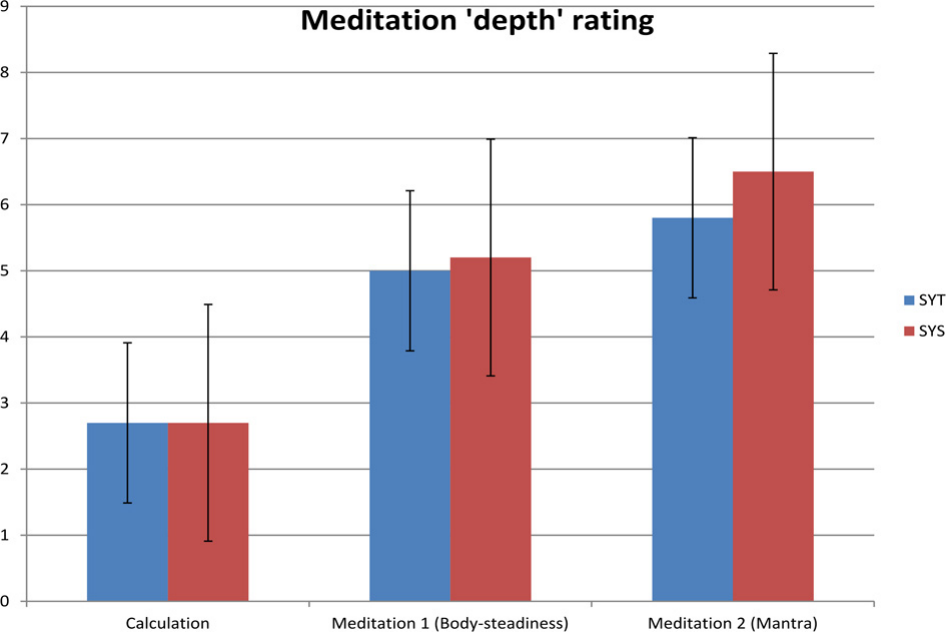
**Subjective ratings of meditation “depth.**”

The subjective reports of “meditation depth” are shown in Figure 1. A low level of “meditation depth” was reported in the non-meditation (Calculation) condition, with increasing depth reported with progression through the meditation conditions. The highest rating for meditation depth was for Meditation 2 (Mantra), although this may include a duration effect. The reported levels of ratings were similar between the groups, but with slightly higher levels for the student group (SYS) than the teacher group (SYT).

### Comparison of groups across conditions

We conducted a single test for CSD differences between SYS and SYT groups including all frequency bands (delta, theta, alpha1, alpha2, beta, and gamma). Voxel by voxel the between group log *F* ratio (equivalent to a *t*-test statistic) was calculated for each frequency band considered simultaneously, resulting in 6239 × 6 comparisons. Following the procedure of Nichols and Holmes (2002) implemented in the KEY Institute eLORETA software package, we applied 5000 random permutations to calculate the distribution of the *F*-max voxel statistic (the maximum *F-*value for the full voxel set). The resulting distribution establishes the threshold for significance of the *F* statistic obtained at individual voxels in a way which controls for the family-wise error rate due to multiple testing. The *p*-values presented below represent the probability of the obtained *F* statistic at each voxel under the null hypothesis while simultaneously correcting for multiple testing.

Tables 1–3 show for each frequency band the region, Brodmann area (BA), Tailairach coordinates and the (absolute) maximum voxel *F* statistic or statistical difference between SYT and SYS groups and total number of significant voxels (two tailed threshold) for Meditation 1 (Body-steadiness), Meditation 2 (Mantra), and the non-meditation condition (Calculation), respectively.

**Table 1:** **SYT > SYS comparison for Meditation 1—(Body-steadiness)**.

**Band**	**Max/min region**	**Lobe**	**Max/min BA**	**Tailairach coordinates**	**Max/min *F*-value**	**No. of voxels *p* < 0.05**
Delta	Inf parietal lobule	Parietal	R40	40, −40, 60	−0.729 ns	
Theta	Inf parietal lobule	Parietal	R40	45, −45, 60	−1.32^**^	490
Alpha1	Precentral gyrus	Frontal	R4	55, −10, 45	−2.01^**^	1827
Alpha2	Inf parietal lobule	Parietal	R1	45, −30, 65	−1.08^*^	30
Beta	Sub−gyral	Temporal	R20	40, −10, −25	1.03*	33
Gamma	Fusiform gyrus	Temporal	R20	40, −15, −30	1.65^**^	1631

* p < 0.05,

** p < 0.01.

**Table 2:** **SYT > SYS comparison for Meditation 2—(Mantra)**.

**Band**	**Max/min region**	**Lobe**	**Max/min BA**	**Tailairach coordinates**	**Max/min *F*-value**	**No. of voxels *p* < 0.05**
Delta	Ant cingulate	Limbic	L32	−10, 35, −5	0.822 ns	
Theta	Inf parietal lobule	Parietal	R40	50, −50, 55	−1.52^**^	613
Alpha1	Precentral gyrus	Frontal	R4	50, −10, 50	−1.97^**^	1676
Alpha2	Sup temp gyrus	Temporal	R22	45, −20, 0	1.10 ns	
Beta	Rectal gyrus	Frontal	L11	−10, 40, −25	1.30^*^	917
Gamma	Insula	Sub−lobar	R13	30, 20, 15	1.92^**^	2092

* p < 0.05,

** p < 0.01.

**Table 3:** **SYT > SYS comparison for control condition (Mental calculation)**.

**Band**	**Max/min region**	**Lobe**	**Max/min BA**	**Tailairach coordinates**	**Max/min *F*-value**	**No. of voxels *p* < 0.05**
Delta	Sup temp gyrus	Temporal	R41	55, −25, 5	−1.72^**^	390
Theta	Sup temp gyrus	Temporal	R22	65, −20, 0	−1.07 ns	
Alpha1	Precentral gyrus	Frontal	R6	45, −10, 40	−1.96^**^	856
Alpha2	Inf parietal lobule	Parietal	R40	50, −50, 55	−1.43^*^	123
Beta	Sub−gyral	Temporal	L20	−45, −10, −25	1.07 ns	
Gamma	Fusiform gyrus	Temporal	R20	40, −15, −30	1.52^*^	85

* p < 0.05,

** p < 0.01. Positive value for F indicates SYT shows higher CSD than STS, negative value for F indicates SYS has higher CSD than SYT.

### Differences between groups on source activation frequency and location in meditation conditions

The two meditation conditions showed a similar pattern, the SYS (intermediate group) having voxels with significantly higher CSD in the lower frequencies (theta, alpha1) and the SYT (advanced group) having voxels with significantly higher CSD in the higher frequencies (beta, gamma). The SYS group had significantly higher CSD voxels in the alpha2 band in Meditation 1 (Body-steadiness) but not Meditation 2 (Mantra). No significant voxel differences were found in either meditation condition in the delta band.

**Figure 2:**
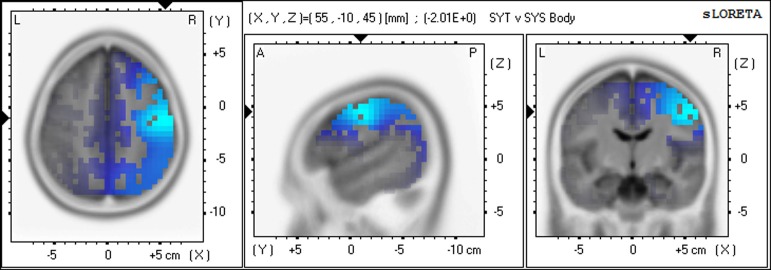
**Differences in CSD between groups in alpha1 band—Meditation 1 (Body-steadiness) Increased alpha1 activity (blue) in SYS compared to SYT in right BA4 (precentral gyrus)**.

**Figure 3:**
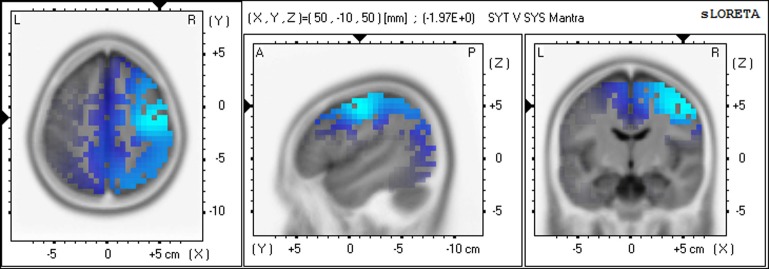
**Differences in CSD between groups in alpha1 band—Meditation 2 (Mantra) Increased alpha1 activity (blue) in SYS compared to SYT in right BA4 (precentral gyrus)**.

For the SYS group compared to the SYT group the greatest number of voxels with significantly higher CSD values were in the alpha1 band, predominantly in the right hemisphere. For both meditation practices, the three Brodmann areas in which the highest alpha1 voxel *F*-values were located were, in rank order of that statistic, right BA4 (precentral gyrus), BA6 (precentral gyrus), and BA3 (primary somatosensory cortex). (See Figures 2, 3).

For the SYT group compared to the SYS group, the greatest number of voxels with significantly higher CSD occurred in the gamma band, predominantly in the right hemisphere. In Meditation 1 (Body-steadiness), the highest voxel statistic values were located in right BA20 (fusiform gyrus), BA21 (middle temporal gyrus), BA13 (insula), BA38 (the anterior pole of the temporal lobe). In Meditation 2 (Mantra), the highest voxel statistic values were located in right BA13 (insula), BA45, and BA47 (inferior prefrontal gyrus). (See Figures 5, 6).

### Differences between groups on source activation frequency and location in calculation condition

**Figure 4:**
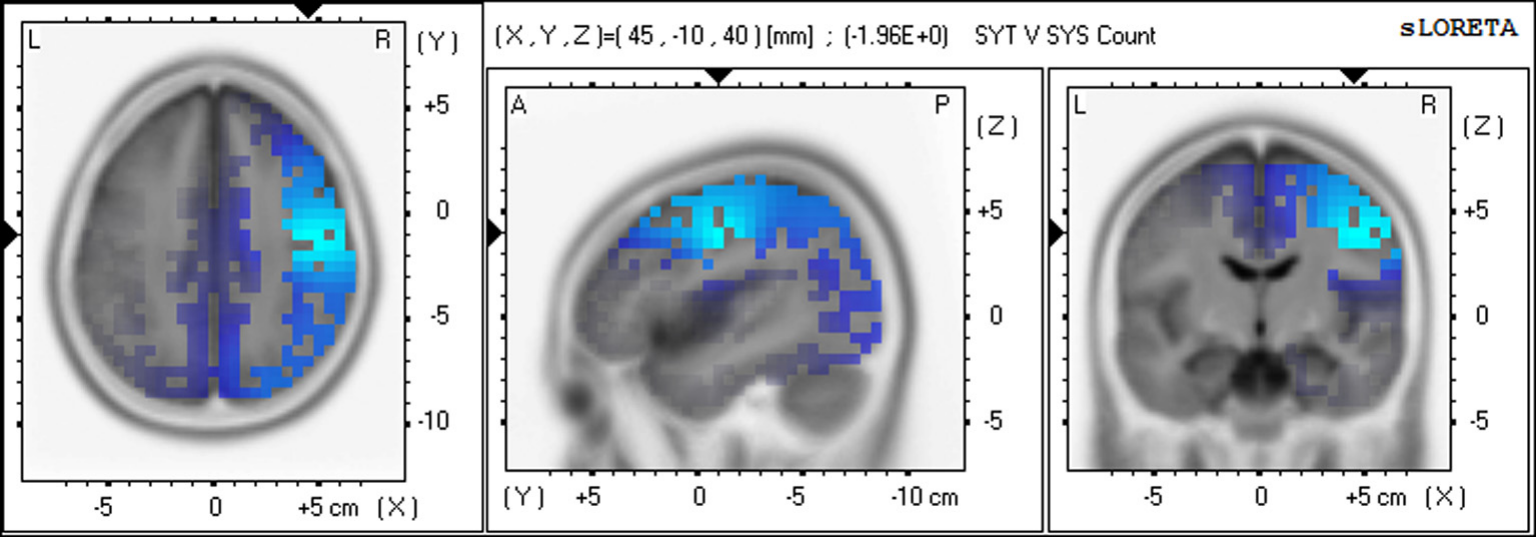
**Differences in CSD between groups in alpha1 band—Non-meditation (Calculation) Increased alpha1 activity (blue) in SYS compared to SYT in right BA6 (precentral gyrus)**.

Significant differences were found for voxels across the delta, alpha1, alpha2, and gamma frequency bands, with the SYS group showing significantly higher CSD voxels than the SYT group in delta, alpha1, and alpha2 bands. In these low frequencies, the locations of significant voxels were entirely in the right hemisphere, the voxels with the highest *F*-values being located in rank order in right BA41, 22, 21 in delta; in right BA6, 4, 3 in alpha1; and in right BA40, 7, 19 in alpha2. (See Figure 4 for alpha1).

In the high frequency gamma band, the SYT group showed voxels with significantly more CSD than the SYS group. Once again these voxels were found entirely in the right hemisphere, the voxels with the highest *F*-values being located in rank order in right BA20, 13, 21. (See Figure 7 for gamma).

### Cortical sources of differences between groups in high and low frequency bands

Across all conditions the number of significant differences was consistently greatest in the gamma band for the high frequencies and the alpha1 band for the low frequencies (highlighted in Tables 1–3 above). In order to examine the specific regional pattern of these high and low frequency group differences, the number of significant voxels in each (right and left) Brodmann Area are presented for each experimental condition in the alpha1 band and gamma bands in Tables 4, 5.

**Table 4:** **Number of significant voxels in each Brodmann Area for each condition in alpha1 band**.

**No. signif voxels**	**Alpha1 band (8–10 Hz)**				**SYS > SYT**	
**BA**	**Calculation**		**Body**		**Mantra**	
	**L**	**R**	**L**	**R**	**L**	**R**
1		9		9		9
2		47		49	2	53
3		59	12	72	17	70
4		54	12	63	13	61
5		12	27	50	32	50
6		170	38	265	84	267
7		130	92	208	113	208
8		33		64	1	76
9		55		84		69
17		8		40		13
18		25		158		48
19		64	6	145	2	98
23		3		6		2
24		1		34	14	44
30		1		18		6
31		12	12	90	27	69
32		1		12	2	20
37				14		3
39		28		58		55
40		143		163	3	149
43				3		
44				3		
45		1		6		1
46				4		
Total	0	856	199	1627	310	1371

Brodmann areas with no voxels omitted from table.

**Table 5:** **Number of significant voxels in each Brodmann Area for each condition in gamma band**.

**No. signif voxels**	**Gamma band (25–42 Hz)**				**SYT > SYS**	
**BA**	**Calculation**		**Body**		**Mantra**	
	**L**	**R**	**L**	**R**	**L**	**R**
4			2	7	4	5
6		1	7	46	9	55
8					4	85
9				22	16	135
10			14	109	133	135
11			101	122	115	122
13		28	51	143	83	79
20		22	56	86	69	50
21		7	40	108	71	45
22		10	34	75	48	27
24			2	3	5	14
25			15	22	16	27
28			10	18	17	18
32			10	24	30	59
34		1	8	16	17	16
35			2	5	3	6
36		1	6	11	9	8
38		9	76	88	76	88
40				6	1	
41				20	10	
42				17		
43			5	12		
44		5	6	28	43	28
45				34	16	34
46				25	13	25
47			97	113	107	113
Total	0	84	542	1162	915	1175

Brodmann areas with no voxels omitted from table.

## Discussion

### Hypotheses 1 and 2

The first prediction that during Yoga meditation student (trainee-teacher) meditators would show greater CSD in the low frequency bands was supported in the theta, alpha1 and alpha2 bands for Meditation 1 (Body-steadiness) and theta and alpha 1 for Meditation 2 (Mantra). In both meditations the number of significant voxels at the lower frequencies was overwhelmingly greatest in the alpha1 band (See Tables 1, 2).

The second prediction that advanced practitioners would show greater CSD in the high frequency bands was supported for beta and gamma frequency bands in both meditation conditions. For the higher frequencies the number of significant voxels was overwhelmingly greatest in the gamma band (See Tables 1, 2). This is the first study to show enhanced gamma band activation in advanced Western meditators practicing in the Yoga tradition, compared to less experienced practitioners.

More broadly the *F-*max voxel values for SYT vs. SYS were negative for all low frequencies (delta to alpha2) in all experimental conditions (including mental calculation) and positive for all higher frequency bands (beta and gamma), (See Tables 1–3). It appears then that patterns of increased (low frequency) EEG activity in Yoga meditation students are consistent with the tradition based expectations of pratyahara (somatosensory withdrawal), evidenced by significantly greater low frequency activity corresponding to inhibition of cortical processing, in those regions mediating external sensory and motor processing. By contrast, patterns of increased (high frequency) EEG activity in advanced Yoga teachers most likely correspond to activity in brain regions recruited in the conscious states of concentration and absorption, which emerge in advanced practitioners.

While the relationship of the neural networks engaged by meditation practice and hypnotic induction remains to be determined it is intriguing to note that a recent study of the neurophenomenology of neutral hypnosis (what some would term trance) found that for those with high hypnotic susceptibility self-reported hypnotic depth correlated significantly with EEG activity measures uniquely in beta and gamma band frequency ranges (Cardeña et al., 2013). At the same time the high (but not low or medium) susceptible group reported spontaneous exceptional experiences of positive affect and/or self-transcendence.

### Hypotheses 3 and 4

**Figure 5:**
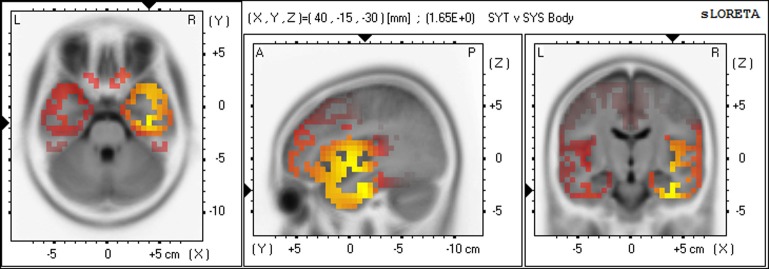
**Differences in CSD between groups in gamma band in Meditation 1 (Body-steadiness) Increased gamma activity (yellow) in SYT compared to SYS in right BA20 (fusiform gyrus)**.

**Figure 6:**
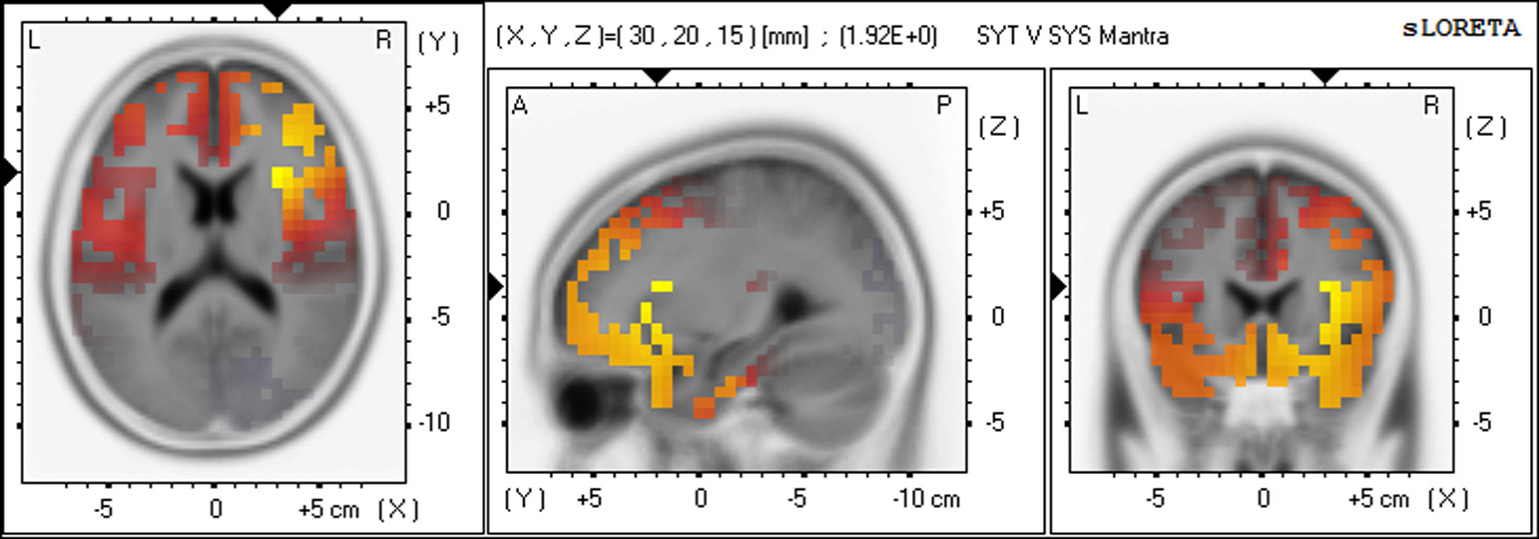
**Differences in CSD between groups in gamma band in Meditation 2 (Mantra) Increased gamma activity (yellow) in SYT compared to SYS in right BA13 (insula)**.

Tables 4, 5 show that for the alpha1 and gamma bands, respectively, only right hemisphere Brodmann Areas show significant differences between SYT and SYS in the non-meditation (counting backwards by four) condition. Looking across the rows of these tables these same regions are also found to have the greatest number of significant voxels in the “Body-steadiness” and “Mantra” meditation conditions (typically with a greatly increased number of significant voxels). We interpret this pattern as support for our third prediction of trait differences in baseline neural activity brought about by long term meditation practice in those regions most engaged by this practice. We further interpret these findings as evidence for a core right-sided (rather than midline) network (or networks if the alpha1 and gamma results are considered separately) that is progressively modulated over the course of Yoga meditation practice. The predominantly right lateralized location of the cortical sources where activation differentiates between the STS and SYT groups can be observed for both alpha1 and gamma in each of the experimental conditions in Figures 2, 3, 5, 6. Thus, in the case of Yoga meditation practice (as distinct from recent studies of mindfulness/Buddhist meditation practices) the midline nodes of the DMN, although included in regions of significant voxel differences, do not appear to be a principal locus of practice-related changes in cortical activity (contradicting the fourth prediction above). Our result also differs from the CSD changes in midline DMN structures recently reported in “concentrative” (breath-focused) meditation with “intermediate level” (mean 4 years) practitioners of unspecified tradition compared to controls (Lavallee et al., 2011). It also suggests an important difference (in advanced meditators) with the neutral hypnotic state in so much as hypnotic induction has been found to reduce (fMRI measured) anterior DMN activity in high hypnotically susceptible participants (McGeown et al., 2009).

**Figure 7:**
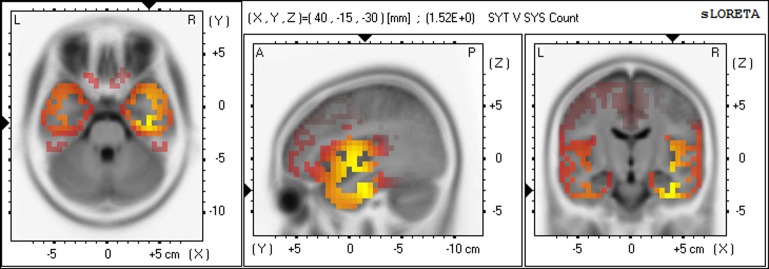
**Differences in CSD between groups in gamma band in Non-meditation (Calculation) Increased gamma activity (yellow) in SYT compared to SYS in right BA20 (fusiform gyrus).**.

### Regional differences in experimental conditions related to Yoga meditation proficiency alpha1 band

In the alpha1 band, significant voxel differences always showed greater activity in the student than the teacher meditators. In all conditions, the most significant voxel *F*-values were always located in the right somatosensory (BA1, 2, 3, 5), motor (BA4) and premotor cortex (BA6). In the mental calculation condition, a core network of right sided regions can be identified. This was comprised, in addition to those regions identified immediately above, of right occipital (BA17, 18, 19), right parietal (BA7, 39, 40) and right superior frontal gyrus (BA8, 9). These same regions remain the core of the most significant proficiency related differences in both meditation conditions. The key changes are an increase in the number of significant voxels recruited in each region and a spread of significant voxels to the homologous left sided Brodmann Areas, while always retaining a strong imbalance toward right sided voxels (see Table 4). Following current models of the functional role of task related topographic differences in alpha band activity, these effects may be interpreted as trained task specific patterns of functional cortical inhibition (Klimesch et al., 2007). Such an interpretation of present alpha1 findings fits remarkably closely with the specific effects of the inhibitory thalamo-cortical mechanism proposed by Austin (2013) to account for states of deep absorption corresponding to Yoga meditation practices. However, in Austin's model these effects would be expected to be greatest in advanced rather than intermediate practitioners.

### Gamma band

In the gamma band, significant voxel differences always showed greater activity in the teacher than the student meditators. In the non-meditation (mental calculation) condition all significant voxel differences were found in the right anterior temporal lobe (principally BA20, 21, 22, 38) and right ventral prefrontal cortex (principally BA13 and BA44). All these regions showed a great expansion of significant voxel counts in the body-steadiness and mantra meditations.

In the “Body-steadiness” meditation, 1704 out of a possible 6239 cortical voxels showed significant proficiency differences and in the “Mantra” meditation this rose to 2090 significant voxel differences. A right sided bias continued to be observed for both meditations but was much less extreme than in the alpha1 band. Additional regions showed significant greater CSD in gamma for the long term meditators in both meditations (principally in BA10, 11, 45, 47) (see Table 5).

This result adds to the converging lines of enquiry regarding the role of meditation-specific increases in gamma band activity in “advanced” practitioners, adding evidence from Western meditators practicing in an integral Yoga spiritual tradition. The teacher group in this study (mean experience 30 years) displayed striking increases in gamma band activity similar to studies of “advanced” Tibetan Buddhist meditators (Lutz et al., 2004) and Western Buddhist Vipassana meditators with mean experience 20 years (Cahn et al., 2010). Although those studies did not estimate the activity of cortical sources, the high level of gamma activity during meditation in advanced practitioners requires widespread synchronization throughout extensive cortico-cortico and cortico-thalamic neural networks (Lutz et al., 2004).

In Meditation 1, the “Body-steadiness” meditation, the most significant voxel differences were located in the right anterior temporal lobe and the insula. This result aligns with that of an advanced Tibetan Buddhist meditator showing increased gamma activity in right mid temporal gyrus (BA21) in the comparisons of sensory-focused “visualization” meditation vs. the verbal-focussed “mantra” meditation and also in a “self-reconstruction” vs. “self-dissolution” meditation (Lehmann et al., 2001). In Meditation 2, the “Mantra” meditation, the most significant sources were located in the right insula and right inferior frontal gyrus. The right insula has been linked with a more detached and objective awareness of interoceptive sensory events (Farb et al., 2007) involved in the shift from “narrative” to “experiential” self-awareness. Deen et al. (2011) identify three distinct clusters of functional connectivity with network hubs in posterior, mid, and anterior insula, respectively, suggesting that a further parcelation of anatomical and function subregions within the insula will be required to fully understand the role (or roles) it plays in Yoga and Buddhist tradition meditative states. It may be of note that a major white fiber tract, the uncinated fasciculis, mediates bidirectional connections between the high level association cortex of the anterior temporal lobe and the inferior frontal gyrus (Von der Heide et al., 2013), the only such structure which continues to develop beyond the age of 30 (Lebel et al., 2008).

### Strengths of this study

A strength of this study is the advanced level of meditation experience in a sample of Western Yoga practitioners following an identical spiritual tradition. All of the “advanced” group were *sannyasin* (initiated) disciples of Swami Satyananda who had spent considerable time in ashrams, both in Australia and India. They were all Yoga teachers. Studies reporting results from large groups of advanced meditation practitioners often combine meditators from different traditions into a single experimental group. Such differences are not considered equivalent by the members of these traditions themselves and any such equivalence must be demonstrated rather than assumed. We have chosen not to make this assumption even at the cost of a lower sample size in order to maintain the validity of our analysis. A further strength was the attention given to preserving the ecological validity of the meditation states attained, the study being conducted in a meditation setting conducive to the participants' usual practice. The intrusion of equipment into this setting was minimized, with the EEG cap and small radio transmitter being the only items in the meditation room.

As with all EEG source analysis methods, eLORETA results are highly susceptible to distortion by artifacts (Pizzagalli, 2007), therefore the quality of data preprocessing is essential to the validity of the results obtained. We adopted a recently developed (Nolan et al., 2010) ICA component rejection procedure for artifact cleaning developed by the Neural Engineering Group at Trinity College Dublin. This is one of a new generation of ICA artifact correction procedures which is guided by a series of objective component parameters rather than the momentary choice of the individual experimenter. We believe the effective utilization of this method for preparing large volumes of clean data was an essential factor contributing to the validity of this research. We strongly recommend the future adoption of this or similar method by related research programs.

### Limitations of this study

Limitations of the study include the small sample size (although that did not prevent significant findings) and the potential confound due to significant age difference between the subject groups. A larger sample size is desirable to enhance the power and generalizability of the current results and would allow the additional possibility of parametric testing (Thatcher et al., 2005). However, the non-parametric statistical mapping method adopted here is well suited to the limitations of the current design and sample size. In fact, one of the worked examples offered by Nichols and Holmes (2002, p. 15) is a PET study with 12 subjects equally divided amongst 2 testing conditions which directly reflects the constraints of the present study. In addition the randomization distribution is known to be overly conservative when the effect is distributed, as occurs in the present results (Troendle, 1995). Therefore, we believe that there is a strong justification for reporting the group effects identified here.

EEG spectral power density changes dramatically with age until early adulthood. From that point changes are slow in healthy aging. Power in lower frequency bands (delta through alpha) decreases throughout this time period (Rossini et al., 2007). The age range of our subjects extends from 30 to 63, so it could be that significantly greater activity in the younger group in these lower bands is due to normal aging rather than years of meditation practice.

A comparison of the effects of normal aging to the presently observed differences between the SYS (younger) and SYT (older) studies of EEG spectral source changes across this part of the lifespan shows that delta decreases in occipital sources (Babiloni et al., 2006), but we only found significant delta differences in one condition (counting) and this was maximal in right BA41 (primary auditory cortex). In normal aging alpha1 and alpha2 decreases in occipital, parietal, temporal and limbic cortices (Babiloni et al., 2006). We found significantly lower alpha1 in occipital and parietal cortex but not in temporal or limbic cortex. Alpha also increases with age in frontal cortex (Basar, 2012) but we found significant alpha *decreases* in superior and middle frontal gyri in the older group and no significant differences in inferior frontal gyri. Once again, the pattern of our findings does not fit with what would be expected if they were due to normal aging.

Despite available data there is no clear evidence for changes due to normal aging through middle age in the high frequency bands. Therefore, we do not consider age differences to be a plausible alternative explanation for those high frequency effects. A confound due to the age difference between the groups fails to account for why the set of BA containing significantly greater low frequency voxel activation in the SYS group forms the complement to the set of BA containing significantly greater high frequency voxel activation in the SYT group. Nor does it explain why a core pattern found in the non-meditation Calculation condition is maintained but greatly expanded in the Body Scan and Mantra meditation conditions. Lutz et al. (2004), in a study with advanced and novice meditators with age means of 49 years and 21 years, found hours of practice, but not age, significantly predicted gamma in their baseline condition.

The additional analysis of age matched subgroups goes some way toward addressing this issue with significant *F*-max voxel findings in both alpha1 and gamma bands located in the same regions as the original group analysis. In this case the further reduced sample size points to the robustness of these effects. Notwithstanding these considerations practice related differences in age will be intrinsic to group membership whenever truly “advanced” (such as the 30 year group in the present study) are compared with medium term practice groups. Now that specific regions of interest have been identified parametric methods which enable statistical control of age effects present a viable option to address this issue in future (larger n) studies.

The density (number) of electrodes in the recording array also places limitations on the resolution of source localization (Pizzagalli, 2007). Dense array recordings are so far limited to laboratory settings, unlike the present study. However, successful validation studies of LORETA, sLORETA and eLORETA use only the original 19 electrodes of the 10–20 system (Pascual-Marqui et al., 2002). Particularly for non-evoked recording conditions, 19 channel recordings are considered adequate and are widely reported for the LORETA family of source analysis methods (Babiloni et al., 2014). By comparison, the current study employed 25 active recording electrodes. A further possible limitation was the fixed sequence of conditions rather than a systematically counterbalanced design. For investigation of deeper meditation states, the benefits of experimental manipulation of conditions and intrusive measuring instruments must be balanced by the need to obtain an authentic meditation experience.

## Conclusion

This study is the first to report enhanced gamma band activation in advanced Western meditators practicing in the (Satyananda) Yoga tradition, compared to less experienced (intermediate) practitioners. It found significant differences in EEG frequency band sources between “experienced” (mean 4 years) and “advanced” (mean 30 years) meditators in two meditation conditions (Body-steadiness and Mantra) and a mental calculation condition. The findings, strongest in alpha1 and gamma bands, consistently support the frequency band hypothesis proposed by Fell et al. (2010) of enhanced low and high frequency band effects, respectively, for intermediate and advanced levels of meditation experience. It further adds a finding of increased gamma band activity in advanced western Yoga meditators to similar findings in advanced Buddhist meditators. In addition, the Body-steadiness meditation condition in the present study appears to have much in common with Buddhist mindfulness of breathing practice. Both practices direct awareness to the felt quality of bodily sensations. The present findings then call for much closer scrutiny of the distinction, at least in the long term, between the functional networks trained by concentrative and mindfulness meditation methods. Those regions which consistently differentiated between the groups in the low and high frequency bands across all meditation conditions showed similar (but reduced) differences in the non-meditation (counting backwards) condition.

This study extends previous EEG findings by estimating cortical gray matter sources for frequency band specific meditation training effects. The cortical loci of high and low frequency band meditation training effects are both specific and distinct. Approximately one third of available cortical gray matter voxels showed significantly greater gamma band activity in mantra meditation in advanced (SYT) than intermediate (SYS) meditators. Despite this, there is little overlap with those regions which show increased alpha1 in the student meditators. In fact the Brodmann areas in which significant voxel differences are found during meditation in the alpha1 (suggesting functional inhibition) and gamma bands (suggesting integration into consciousness) form almost absolute complement subsets of the full set of cytoarchitecturally defined gray matter regions. We propose that selective inhibition of a right lateralized network comprising visual, somatosensory and body-world self-representations corresponds to the earlier stages of sensory withdrawal and stripping away of the “outer” onion-like layers of self as described in traditional Yoga literature. The subsequent emergence of conscious states specific to advanced practitioners requires both the disengagement from these self-world representational systems and the development of widespread gamma synchronization throughout anterior temporal and ventral prefrontal cortical regions extending from a right sided core network incorporating anterior temporal lobe and insula.

### Conflict of interest statement

The authors declare that the research was conducted in the absence of any commercial or financial relationships that could be construed as a potential conflict of interest.

## Footnotes

^1^In the present study Buddhist and Yoga meditation practices are neither assumed to be equivalent nor different at a neurophysiological and/or psychological level, rather this is something to be established by empirical investigation.

^2^The research reported here formed part of the Ph.D. thesis of the first author, supervised by the second and third authors. Following the framework of Cahn and Polich (2006) the thesis sought to determine expected trait (length of practice) effects, state (meditation vs. non-meditation) effects and state by trait interaction effects of Yoga meditation practice in EEG cortical source activity. Specific trait expectations (see above) were principally based upon the work of Farb et al. (2007) (for cortical regions) and Fell et al. (2010) (for frequency bands) and it is the results pertaining to these hypotheses that are reported here. It was expected that state effects would be found in higher high (i.e. gamma) frequency source activity in cortical regions specialized in processing the different contents of specific meditations (e.g., bilateral parietal and somatosensory cortex for body scan and left inferior frontal gyrus for mantra) during those specific meditations as compared to the non-meditation condition. Following the person by situation interactionist approach in personality psychology it was expected that state by trait interaction (as distinct from additive) effects would be highly significant (and informative) although the specific form of those effects was not predicted. Neither the expected state nor state by trait interaction effects were found. We consider these null findings to be highly informative (although beyond the scope of the current report) and they have reorientated our perspective to focus on the understanding of trait changes arising over the long term time course of meditation practice.
